# Construct-construct “rail technique” decreases screw strain during spinal deformity corrective maneuvers: a mechanical analysis

**DOI:** 10.1007/s43390-025-01079-y

**Published:** 2025-03-28

**Authors:** Alekos A. Theologis, Jason DePhillips, Nathaniel A. Myers, Jonathan M. Mahoney, Brandon S. Bucklen

**Affiliations:** 1 Department of Orthopaedic Surgery, University of California - San Francisco (UCSF), 500 Parnassus Ave, MUW 3rd Floor, San Francisco, CA 94143 USA; 2 Scientific Affairs, Globus Medical Inc., Audubon, PA USA; 3 Department of Bioengineering, University of Pittsburg, Pittsburg, PA USA

**Keywords:** Spinal deformity, Correction techniques, Cantilever bending, Segmental compression, 3-Column osteotomy

## Abstract

**Purpose:**

To compare screw strains adjacent to a simulated spinal osteotomy between segmental compression (SC) and cantilever bending (CB) to SC and CB performed over a construct-to-construct lateral accessory rod (“rail”).

**Methods:**

10 PCF foam blocks were instrumented with 6 polyaxial pedicle screws, each with a linear strain gage. SC and CB were performed over a traditional construct (midline rods) or over a construct-to-construct lateral accessory rod. Real-time screw strains were collected and peak strains were reported and compared between corrective techniques.

**Results:**

Strains in screws closest to the osteotomy were significantly less during “rail” compression compared to traditional SC. Maximum screw strains were significantly lower during “rail” SC (*p* < .001) and CB (*p* = 0.003) compared to traditional SC and CB, respectively. Total screw strain was more evenly distributed over all 6 screws during “rail” compression and CB compared to traditional techniques, which concentrated strain at individual screws adjacent to the osteotomy.

**Conclusions:**

Performing SC and CB across an accessory construct-to-construct lateral (“rail”) rod resulted in significantly lower strain on individual pedicle screws adjacent to a simulated spinal osteotomy compared to traditional SC and CB. As such, the “rail” may lessen risk of screw pull-out and screw plow during maneuvers to correct spinal deformities. Future work focused on building upon this controlled study in cadaveric specimens will be important to validate these findings in more clinically relevant scenarios.

## Introduction

Spinal osteotomies have become widely accepted posterior-based spinal correction techniques for treatment of adult spinal deformity (ASD) [1–3]. Despite various permutations in techniques and definitions, they can be surgically complex and associated with relatively high rates of neural deficits, particularly related to instrumentation, decompression, and spinal realignment during osteotomy closure [4–6]. The most common maneuvers used for closure and correction across an osteotomy are segmental compression (SC) and cantilever bending (CB). SC takes place when compression is applied to pedicle screws adjacent to the osteotomy, while CB occurs when a rod is under-contoured in the sagittal plane, secured cranial or caudal to the osteotomy, and then pushed down across the osteotomy, resulting in shortening of the posterior column and lengthening of the anterior column [7]. Both maneuvers require a delicate balance between the desired amount of deformity correction and the stresses placed on the bone–screw interfaces by the corrective forces. Inherent to the feasibility and the safety of each technique is the adequacy and the robustness of fixation cranial and caudal to the osteotomy. As the forces to correct deformities can be quite significant, screws adjacent to an osteotomy are prone to loosening, pull-out, and/or plow within the pedicle, resulting in inability to achieve desired correction, construct failure, and possibly neural injury. Frequency of these complications varies (2–15%) [2, 8–10], with the more prevalent occurrence in patients with poor bone quality.

Aiming to limit the aforementioned concerns and minimize bone–screw interface strains across a spinal osteotomy during deformity correction, a construct-to-construct concept was developed and coined the “rail technique” by Theologis et al*.* [11, 12]. This technique utilizes laterally based accessory rods (“rails”) that cross a spinal osteotomy through which SC and CB can occur. As all deformity corrective forces are applied along the “rails”, en-bloc movement of the cranial and caudal spinal segments relative to each occurs across the osteotomy. This provides the following key advantages from a safety and deformity correction stand-point: (1) highly controlled compression, thus minimizing risk of translation; (2) asymmetric compression, thus aiding in correction of concomitant sagittal and coronal deformities; and (3) minimal bone–screw interface forces that decreases the risk of peri-osteotomy screw failure [13–15].

While these benefits have been observed clinically [11, 12], the mechanical advantages of the “rail technique” have not been directly evaluated relative to traditional corrective techniques. Therefore, the purpose of this study is to compare the mechanical behavior of traditional SC to SC across the “rail” as well as to compare the mechanical behavior of traditional CB to CB over the “rail”. The study hypothesis is that the “rail technique” will help mitigate the amount of strain induced on screws surrounding the simulated osteotomy site compared to traditional correction maneuvers.

## Methods

Six polyaxial pedicle screws (5.5 × 45 mm; CREO Stabilization System, Globus Medical, Inc., Audubon, PA) were instrumented with linear strain gages (KFH-03-350-C1-11L3M3R, Omega Engineering, Inc., Stamford, CT). Screw threads were removed directly below the head of each pedicle screw to provide a smooth cylindrical surface for mounting the strain gages. Gages were mounted lengthwise, parallel to the axis of the screw, in order to optimize strain measurements. Once mounted, each gage was layered by a coating of 2-part epoxy (LOCTITE® EA E-120HP, Henkel Adhesives, Bridgewater, NJ) to protect the mechanical and the electrical integrity of the gage.

Screws were then inserted into 10 PCF (0.16 g/cm^3^) polyurethane foam blocks (Sawbones, Vashon, WA), conforming to ASTM standard F1839-08 [16], with care taken to ensure the gage was centered on the axis of the rod. 10 PCF foam was used to represent osteoporotic bone and provide an environment for a controlled mechanical study that eliminated variation of cadaveric specimens. Foam blocks are commonly used in orthopedic device testing as they allow for reproducible and standardized conditions, enabling proof-of-concept testing for novel testing technologies. Screws were placed sequentially starting with screw 1 as the most cranial and screw 6 as the most caudal. After screws were inserted into a foam block, they were tested 7 times for each of the surgical strategies listed below (compression and cantilever bending with the “rail” technique and traditional techniques), and subsequently removed. The testing order of surgical technique was randomized to ensure a balanced evaluation of both techniques while accounting for potential effects of repeated testing on screw purchase. For example, for the compression experiments, the “rail technique” was performed first in four samples (*n* = 4), while the traditional technique was performed first in three samples (*n* = 3). After the initial technique, the alternate technique was performed on the same block without removing the screws. Each screw was configured into a quarter Wheatstone bridge and re-calibrated after each test. During all tests, real-time screw strains were collected at all six screws. Strain data acquisition was done using a multi-channel signal-conditioning amplifier (Model 5100B, Vishay Precision Group, Raleigh, NC) interfaced with a personal computer.

### Compression

**Fig. 1:**
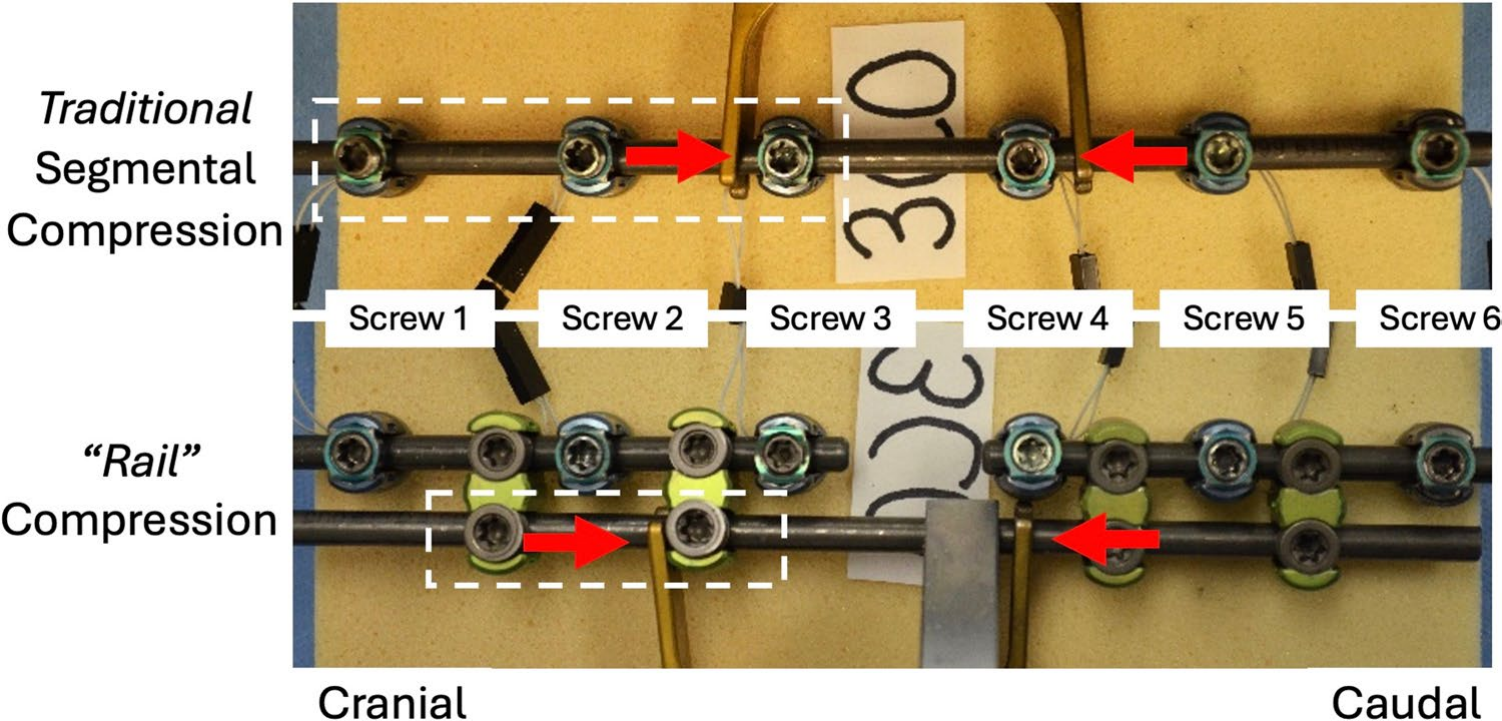
Images of traditional segmental compression (SC) (top row) in which the cranial three screws, highlighted by the white box, remain loose during compression (top row); and compression across the “rail” in which the cranial W-connector’s set screws on the lateral accessory/”rail” rod, highlighted by the white box, remain loose during compression (bottom row)

For traditional SC, a primary 5.5 mm titanium rod was placed in all screws and locking caps were tightened on all screws caudal to the osteotomy (Fig. 1a). A parallel compressor was placed on the primary rod and compression was applied between screws 3 and 4 (Fig. 1a). For SC across the “rail”, separate 5.5 mm titanium primary/midline rods were placed above and below the simulated osteotomy and all locking caps were final tightened (Fig. 1b). An accessory 5.5 mm titanium rod (“rail”) was then attached laterally to the two primary/midline rods via 4 W-connectors (open up-open up) (CREO, Globus Medical, Inc). The locking caps were final tightened on the “rail’s” 2 caudal W-connectors. Because the parallel compressor could not span the length of the W-connectors across the simulated osteotomy, a vise-style rod gripper was first attached to the accessory rod in close proximity to the simulated osteotomy and then the parallel compressor was placed on the accessory rod and compression was applied between the cranial W-connector and the rod gripper (Fig. 1b).

Two compression tests were applied using the parallel compressor: (1) a stepwise compression from one to five instrument notches; and (2) a compression test with a quick force up to a maximum grip strength. Maximum grip strength was defined as the peak force exerted by the dominant hand of the test operator when compressing the constructs. The same test operator performed all measurements to minimize variability. For stepwise compression, a linear regression data analysis was run to analyze screw strain related to the amount of applied compression. For the quick force compression test, statistical analysis was performed on the peak strain reported for screws 1–6.

### Cantilever bending

**Fig. 2:**
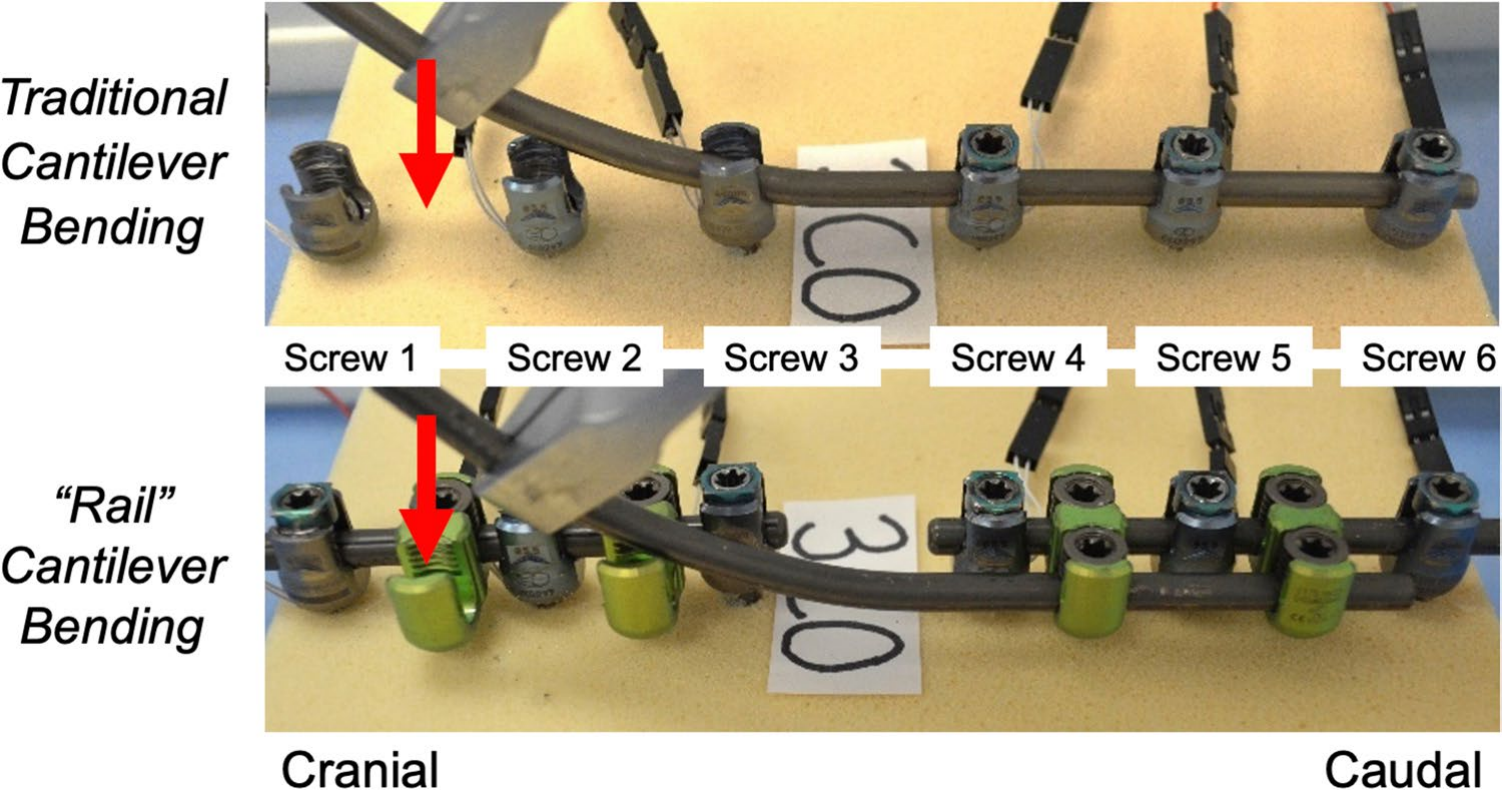
Images of traditional cantilever bending (CB) in which the rod is bent down (red arrow) into the three cranial screws above the simulated osteotomy (top row); and CB over the “rail” in which the lateral accessory rod is bent down (red arrow) into the two cranial W-connectors laterally above the simulated osteotomy (bottom row)

For traditional CB, a pre-bent 5.5 mm titanium rod was placed and secured with tightened locking caps on all screws caudal to the simulated osteotomy (Fig. 2a). This traditional CB construct then underwent a total of 3 tests (each with *n* = 7) based on how many “receiving” screws cranial to the osteotomy were engaged with the rod during CB:

1. “Best-case” scenario: CB with set screws placed on screws 1, 2, and 3 (*S*1 + *S*2 + *S*3).
2. “Worst-case-1” scenario: CB with set screws placed on screws 2 and 3 (*S*2 + *S*3).
3. “Worst-case-2” scenario: CB with set screws placed only on screw 3 (*S*3).

These “worst-case” bending scenarios were selected to represent situations in which securing the rod to multiple points of fixation may not be feasible during the corrective maneuver and or there is rod “spring-back” (i.e., grasp of the rod is lost, screws can’t be engaged, etc.), causing the corrective technique to rely on only one or two “receiving” screws to be anchors for the cantilevered rod, therefore increasing risk of screw failure.

For CB over the “rail,” primary/midline rods (not contoured) were placed above and below the simulated osteotomy and all locking caps were tightened (Fig. 2b). A 5.5 mm titanium rod (“the rail”) was then bent into lordosis and attached and secured laterally to the midline rod below the simulated osteotomy via 2 W-connectors, as previously described by Collins et al. [11]. This was then followed by 2 different CB maneuvers using the “rail” (each with *n* = 7) based on how many of the W-connectors cranially were engaged and secured to the rod during CB:

1. “Best-case” scenario: rod bent down into both W-connectors cranial to the simulated osteotomy (*W*1 + *W*2).
2. “Worst-case” scenario: rod bent down into only the one W-connector closest to the simulated osteotomy (*W*1).

For all CB maneuvers, a vise-style rod grip was attached to the pre-contoured rod and manual force was applied until the rod was seated in the respective cranial screw/W-connector. During CB maneuvers, real-time screw strains were collected at all 6 screws and peak strains were reported.

### Statistical analysis

Statistical analysis was performed using IBM SPSS^®^ Statistics (SPSS^®^ v22, IBM Corp., Armonk, NY, USA). An independent t-test was used to determine significant differences in screw strain between traditional SC and “rail” compression. A one-way ANOVA was used to determine significant differences in screw strain between each of the five CB maneuvers. A statistically significant difference was defined as *p* < 0.05.

## Results

### Compression

**Fig. 3:**
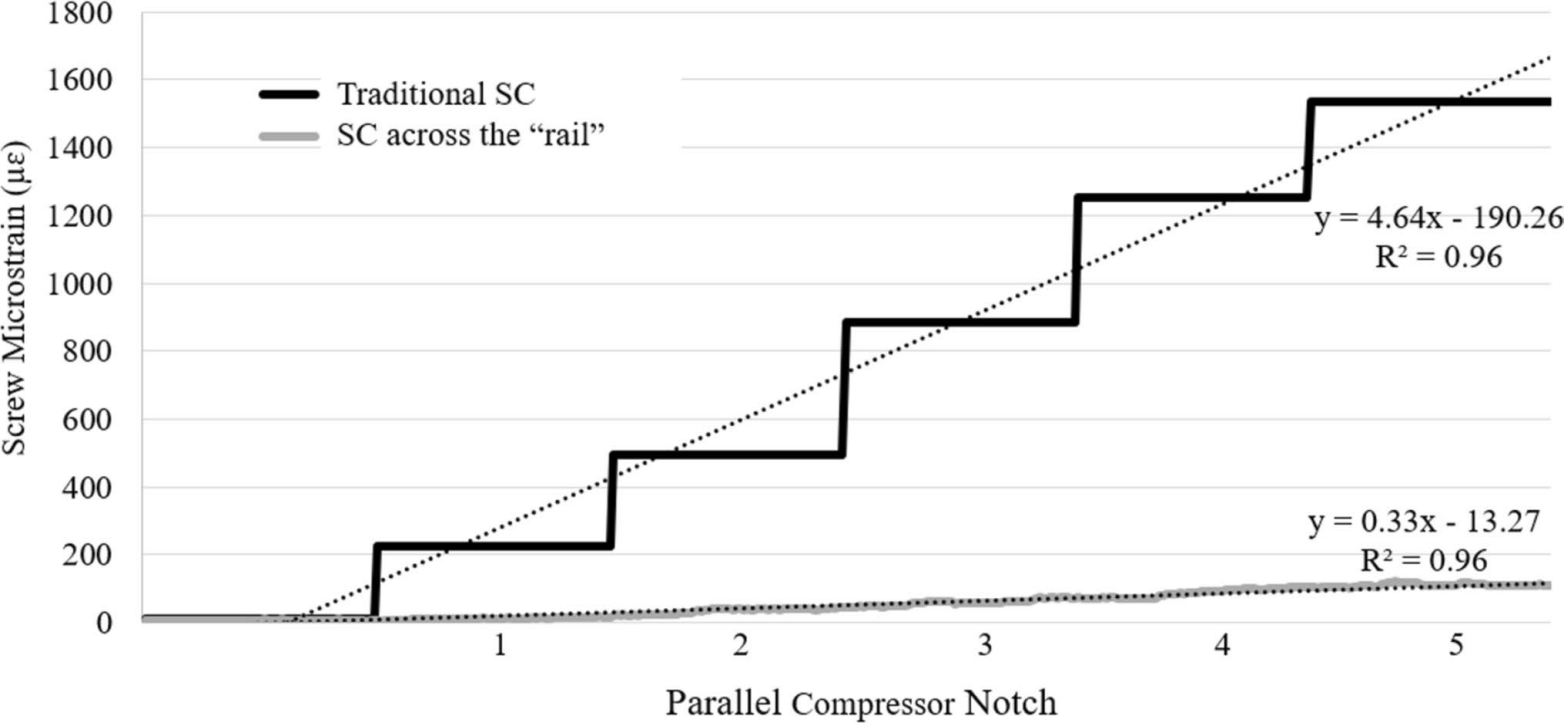
Strain data on screw 3 (adjacent to the simulated three-column osteotomy) during incremental compression using the notches of the parallel compressor for traditional segmental compression (black line) and compression across the “rail” (gray line)

**Fig. 4:**
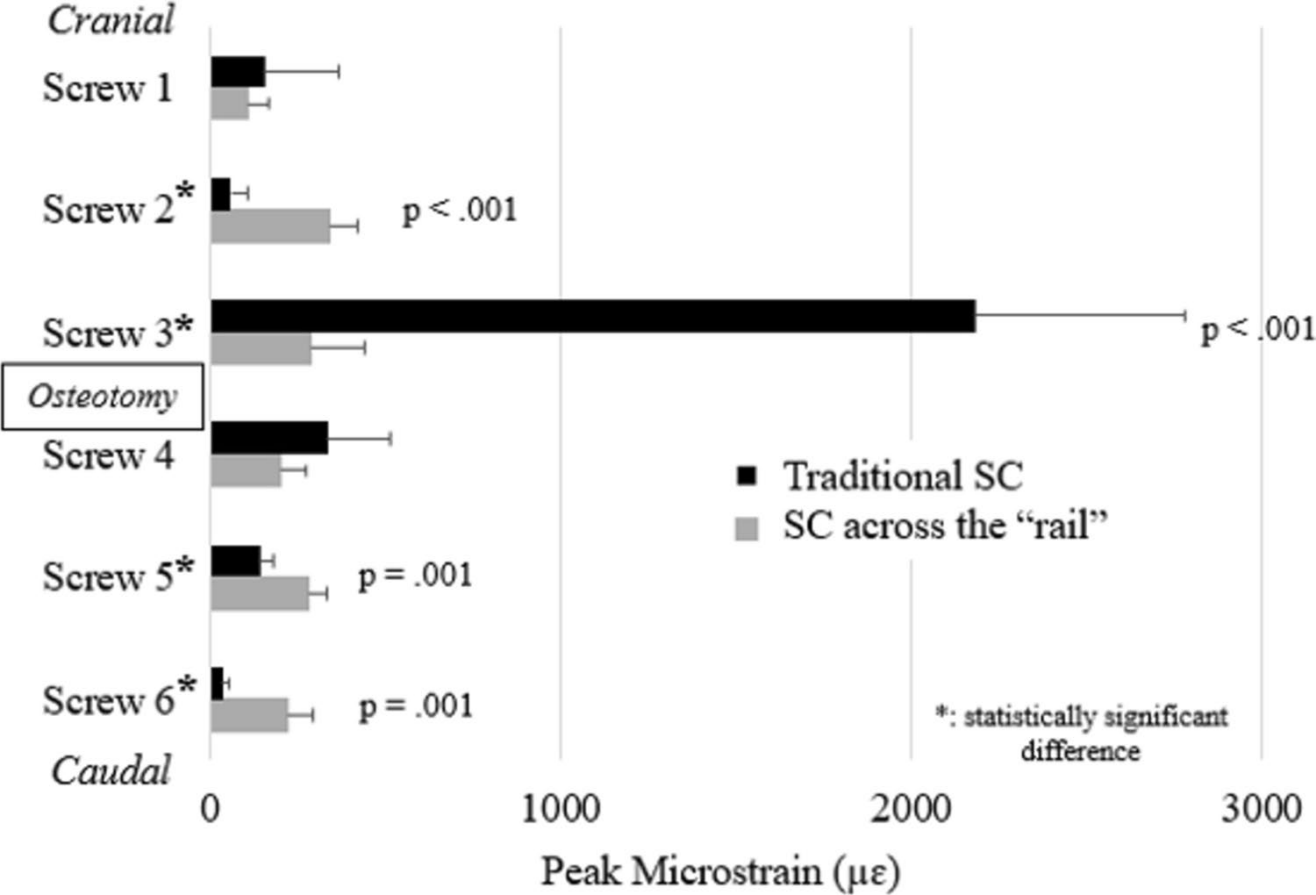
Relative strains for each screw during traditional SC (black bars) and “rail” compression (gray bars) using a maximum grip on the parallel compressor

For the 5-notch compression test, maximum screw strain occurred at screw 3 during traditional SC and “rail” compression. During traditional SC, the rate of strain increase in screw 3 was 1,300% higher compared to “rail” compression (Fig. 3). For the quick force compression test, strain on screw 3 was significantly greater during traditional SC (2184 ± 645.7 µɛ) than “rail” compression (288.6 ± 162.2 µɛ) (*p* < 0.001). While screws away from the osteotomy had significantly greater strains during “rail” compression than traditional SC, absolute values were relatively small (screw 2—344.1 ± 80.2 µɛ *v.* 60.9 ± 52.9 µɛ, *p* < 0.01; screw 5—277.7 ± 63.1 µɛ *v.* 143.4 ± 43.4 µɛ, *p* = 0.01; screw 6—221.8 ± 79.3 µɛ *v.* 37.4 ± 17.4 µɛ, *p* = 0.01**)** (Fig. 4). The screw with the greatest average screw strain in “rail” compression (screw 2: 344.1 ± 80.3 µɛ) had an 84% lower strain than the screw with the greatest average screw strain in traditional SC (screw 3: 2184 ± 645.7 µɛ) (Fig. 4). Additionally, screw strain was more evenly distributed across all 6 screws during “rail” compression (screw 1—7%, screw 2—24%, screw 3—20%, screw 4—14%, screw 5—19%, screw 6—15%) compared to traditional SC in which the majority of the strain (75%) was felt by one screw (#3) (Fig. 4).

### “Best-case” cantilever bending

**Fig. 5:**
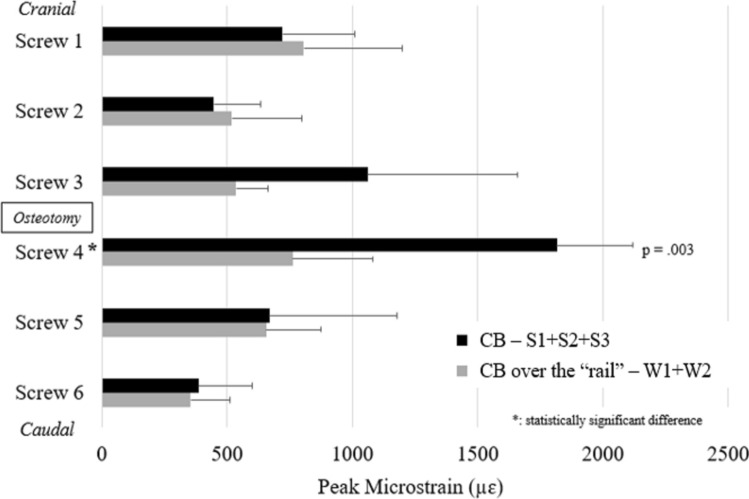
Relative peak strain on each of the 6 screws during “best-case” cantilever bending (CB) scenarios for traditional CB (black bars) and “rail” CB (gray bars)

For “best-case” scenarios, screw 4 had significantly lower strain during CB over the “rail” (762 ± 346.1 µɛ) compared to traditional CB (1817 ± 327.1 µɛ) (*p* = 0.003). This represented a 58% decrease in strain on screw 3 during “rail” CB. Additionally, screw strain was more evenly distributed across all 6 screws during “best-case” CB using the “rail” (screw 1—22%, screw 2—14%, screw 3—15%, screw 4—21%, screw 5—18%, screw 6—10%) compared to “best-case” traditional CB in which strain was more concentrated on the 2 screws adjacent to the simulated osteotomy (screw 3—21%; screw 4—36%) relative to the screws further away from the osteotomy *(*screw 1—14%; screw 2—9%; screw 5—13%; screw 6—8%) (Fig. 5).

### “Worst-case” cantilever bending

**Fig. 6:**
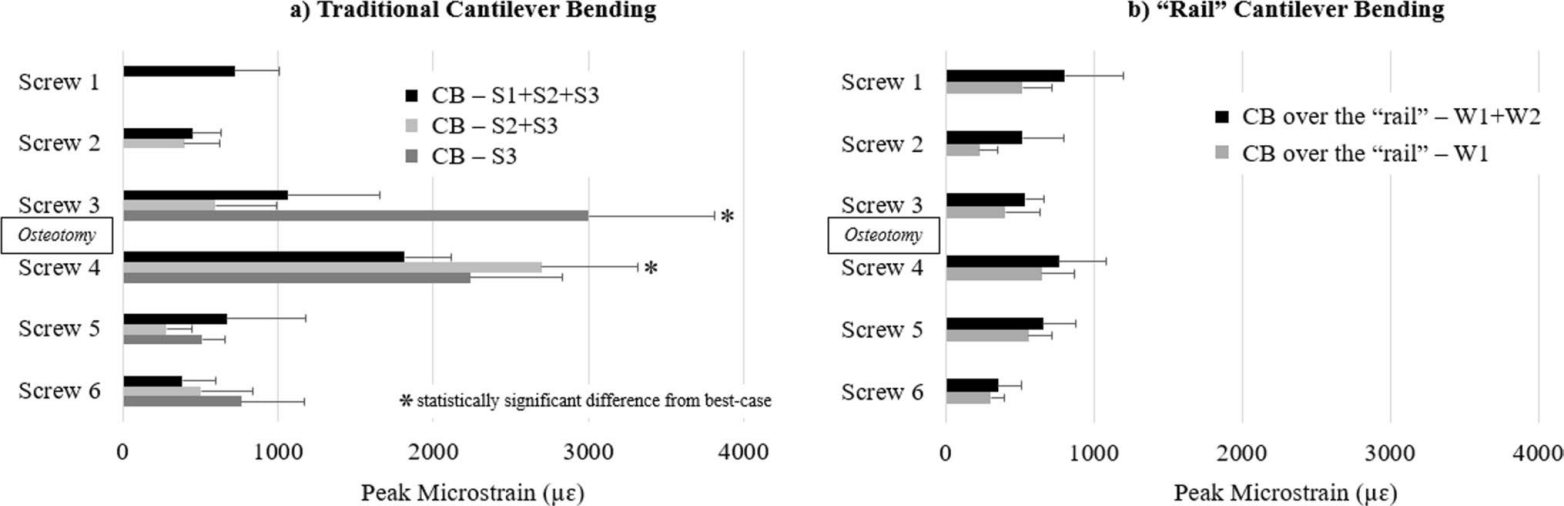
Comparison of screw strains between the “best-case” and “worst-case” cantilever bending (CB) scenarios using a) traditional CB and b) “rail” CB

For traditional CB techniques, the screws adjacent to the simulated osteotomy (#3 and #4) had significantly higher strains during “worst-case” scenarios (i.e., when only one or two of the “receiving” screws were engaged and secured to the rod) (*p* < 0.05). Specifically, screw 4’s strain (2698 ± 672 µɛ) was noted to be 48% greater when only 2 proximal screws (*S*2 + *S*3) were engaged during traditional CB compared to “best-case” scenario in which all three proximal screws were engaged (*S*1 + *S*2 + *S*3). Additionally, screw 3 had the highest strain (3000 ± 877 µɛ) when it was the only receiving screw (*S*3) during the traditional CB maneuver (Fig. 6). This strain represented a 65% increase compared to when all three proximal receiving screws were engaged (*S*1 + *S*2 + *S*3) and an 11% increase compared to when two proximal receiving screws were engaged (*S*2 + *S*3) (Fig. 6).

**Fig. 7:**
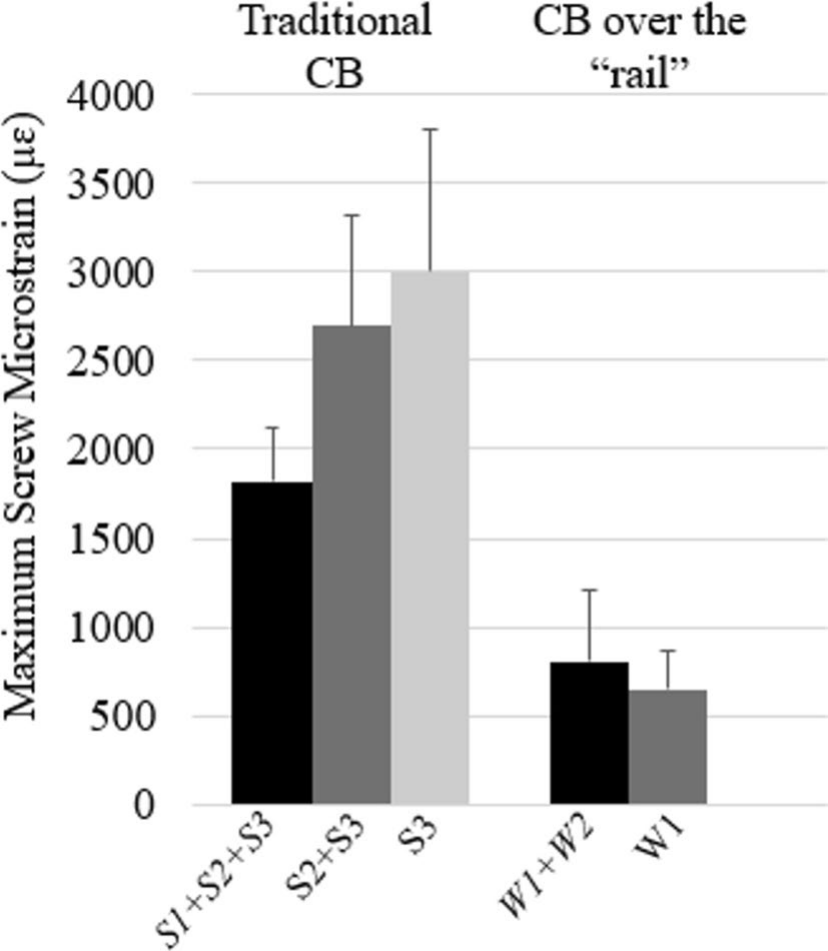
Maximum screw strain during “best- and worst-case” scenarios: CB—*S*1 + *S*2 + *S*3 (screw 4), *S*2 + *S*3 (screw 4), and S3 (screw 3); CB over the “rail”—*W*1 + *W*2 (screw 1) and W1 (screw 4)

For “rail” CB, there were no differences found in screw strain when the rod was engaged/secured into only one (“worst-case”) or both (“best-case”) of the “rail’s” receiving W-connectors (Fig. 6b). During “worst-case” scenario for “rail” CB, the maximum screw strain was observed on screw 4 (650 ± 237 µɛ), which was significantly lower than the maximum screws’ strains measured during “worst-case” scenarios for traditional CB [76% lower than screw 4 during traditional CB when only two proximal receiving screws were engaged (*S*2 + *S*3) and 78% lower than screw 3 when only screw 3 was engaged during traditional CB (*S*3)] (Fig. 7).

## Discussion

The goal of this study was to use a model simulating osteoporotic bone and a reproducible mechanical environment to evaluate screw strains during traditional corrective spinal deformity surgical techniques (segmental compression and cantilever bending) and compare them to SC and CB performed over a construct-to-construct lateral accessory rod (“rail”) across a simulated spinal osteotomy. There were 3 major findings to this study: (1) screws closest to the osteotomy had strains significantly less during SC and CB over the “rail” compared to traditional SC and CB; (2) maximum screw strains were significantly lower during “rail” SC (84%) and CB (58%) compared to traditional SC and CB; and (3) total screw strain was more evenly distributed over all screws during “rail” compression and “rail” CB compared to traditional techniques, which concentrated strain at individual screws adjacent to the simulated osteotomy. These results provide unique initial insights into the mechanical behavior of a relatively novel surgical technique to correct spinal deformities, which to date has previously relied on clinical case reports to showcase its utility.

In the first clinic report of its use, Theologis et al*.* [12] presented a case of a 65-year-old female with osteoporosis who underwent an uncomplicated revision C2-T10 posterior spinal instrumented fusion (PSIF) with shortening across a T4 vertebral column resection (VCR) using the “rail technique” to correct a junctional failure/kyphosis at T4 above a prior T5-pelvis PSIF*.* More recently, Collins et al*.* [11] presented a surgical video demonstrating use of the “rail technique” across a T9 VCR to correct a kyphotic deformity above a prior T10-pelvis PSIF. While both these reports involved deformity correction across a 3-column osteotomy, the ability of the “rail technique” to facilitate highly controlled shortening and concomitantly minimize bone–screw interface forces [13–15] lends support for its use across short-segment areas of deformity that are corrected by posterior-column osteotomies. For example, in 2023, Sarmiento et al*.* [17] reported the safe and successful correction of a multi-planar spinal deformity (85° left lumbar curve with an apex at L1–2; focal kyphotic deformity of 86° from T11–L3) using a similar construct-to-construct biplanar cantilever technique through T11–L4 Ponte osteotomies and a T9–L4 PSIF.

The ultimate utility of the “rail technique” achieved in clinical practice can be attributed to the “rail” rod effectively allowing en bloc shortening across any spinal osteotomy in which multiple spinal units/segments above and below the osteotomy site move together rather than segmental correction in which the required corrective forces are primarily concentrated on individual screws, particularly the screws adjacent to an osteotomy site. As CB relies on engagement of multiple points of fixation to decrease screw strains, we explored “worst-case” cantilever bending scenarios to represent surgical situations when the rod may only be secured to one or two screws on one side of an osteotomy, increasing the risk of screw failure. Noteworthy is that cantilever bending over the “rail” proved to significantly reduce screw strain compared to traditional CB in “worst-case” scenarios, which is secondary to the fact that CB across the “rail” does not require any forces/stresses to be placed directly on any screw, which in turn protects the integrity of all bone–screw interfaces. By distributing necessary corrective force across multiple spinal units and minimizing strain placed on the screws adjacent to an osteotomy, the “rail technique” used in this study demonstrated the potential to lessen the risk of screw pull-out, screw plow, and associated pedicle fractures when correction maneuvers are applied. While this is important for all deformity corrections, it is most necessary in patients who have poor bone quality as any loss of fixation in these settings may prevent safe or achievable deformity correction [18–21]. Given the findings of this study and aforementioned mechanical and safety benefits, the authors of this study use the “rail technique” for all thoracic corpectomies/3-column osteotomies to correct focal deformities secondary to tumor, infection, fractures, and proximal/distal junctional pathology. Another common use of the “rail technique” is for situations in which deformity correction is trying to be achieved using multi-level posterior-column osteotomies across a short-segment of non-rigid deformity (i.e., proximal junctional kyphosis above a prior T3-pelvis posterior instrumented fusion requiring extension to C2 with multi-level Ponte osteotomies from C7–T3).

Biomechanical assessments of different surgical strategies to stabilize spinal osteotomies are quite common [22–24]. The entirety of these prior studies evaluated instrumentation constructs in their “final” form in a static manner. While several prior studies have evaluated the biomechanics of the spine in a dynamic fashion, they were on native/non-instrumented spines. Our study uniquely evaluated stresses on pedicle screws in a dynamic environment, specifically during specific surgical corrective techniques. As we are not aware of prior studies that have used this methodology, we hope that our study will facilitate additional studies that evaluate (bio)mechanics of specific surgical corrective strategies in other common spinal procedures.

The findings of this study should be considered in the context of its limitations. The first limitation is that the study environment provided by the polyurethane foam blocks does not account for the in vivo conditions and variability seen in the clinical application of these surgical techniques as 10PCF foam blocks do not fully replicate the heterogeneity and anisotropy of cadaveric or in vivo bone. While the 10PCF foam failed to simulate the cortical shell of the pedicle, which may impact the mechanical performance of each screw, use of polyurethane foam blocks is an accepted model to represent cadaveric bone under similar testing [25–28]. Repeated testing performed on a single bone block was also a limitation of this study. Additionally, as the mechanical construct in this study is unilateral, it is not completely representative of a true surgical setting in which bilateral rods may be used for stabilization of an osteotomy during deformity correction. While the presence of bilateral rods spanning the osteotomy site (i.e., one acting a stabilizing rod and the second being used as the correction rod) may affect the stresses experienced by the screws during deformity correction, this likely will not change the *relative* results between traditional techniques and “rail” corrective maneuvers given the “rails” can be set-up bilaterally to provide similar provisional stability to bilateral traditional midline rods and because traditional SC and CB strategies will still inherently result in greater concentrated corrective realignment forces on individual screws. Another possible perceived limitation is that the screws, rods, instrumentation, and model were provided by a single spine implant company/manufacturer. However, *none* of the instrumentation and implants used to create the “rail” technique is proprietary to the individual spine company. That performing the “rail” technique is agnostic to implants (i.e., the method is irrelevant to spinal company) and that the spine company does not benefit commercially from this technique should lend validity to the results and minimize potential perceived industry bias. An additional limitation is that the recorded strain magnitudes in this study are a function of the instrumented screws’ lengths, diameters, and material. For example, a screw diameter of 7.5 mm may experience less strain than the 6.5 mm or 5.5 mm diameter due to the superior mechanical strength of a larger screw. Similarly, variations in material properties, such as modulus of elasticity, could influence strain. While these factors may directly affect the magnitude of screw strain, we believe that the mechanical phenomenon/pattern elucidated in this study is generalizable across different configurations. Future studies may systematically explore these variations as well as assess different surgical strategies (i.e., *in-situ* bending) and varying surgical construct lengths and different rod diameters to validate the findings in broader clinical contexts. While the study results should be considered mechanically sound and a unique addition to the literature regarding surgical strategies to correct spinal deformities, future work is focused on building upon this controlled study and acquiring data in cadaveric specimens with the goal to validate these findings in more clinically relevant scenarios.

## Conclusions

In this simulated model of a spinal osteotomy, performing compression and cantilever bending across an accessory rod (“rail”) that connects to instrumentation above/below the osteotomy resulted in significantly lower strain on individual pedicle screws adjacent to the osteotomy compared to traditional SC and CB. As such, the “rail” may lessen risk of screw pull-out and screw plow during maneuvers to correct spinal deformities. While cadaveric models are needed to validate these results, this study’s mechanical findings are sound and help corroborate the previously purported clinical advantages of the construct-to-construct “rail technique” for correction of spinal deformities.

## Data Availability

The data that support the findings of this study are available from the corresponding author, AAT, upon reasonable request.
